# Characterization of a calcium/calmodulin-regulated SR/CAMTA gene family during tomato fruit development and ripening

**DOI:** 10.1186/1471-2229-12-19

**Published:** 2012-02-13

**Authors:** Tianbao Yang, Hui Peng, Bruce D Whitaker, William S Conway

**Affiliations:** 1 Food Quality Laboratory, Plant Science Institute, USDA-ARS, Beltsville 20705, MD, USA

## Abstract

**Background:**

Fruit ripening is a complicated development process affected by a variety of external and internal cues. It is well established that calcium treatment delays fruit ripening and senescence. However, the underlying molecular mechanisms remain unclear.

**Results:**

Previous studies have shown that calcium/calmodulin-regulated SR/CAMTAs are important for modulation of disease resistance, cold sensitivity and wounding response in vegetative tissues. To study the possible roles of this gene family in fruit development and ripening, we cloned seven *SR/CAMTAs*, designated as *SlSRs*, from tomato, a model fruit-bearing crop. All seven genes encode polypeptides with a conserved DNA-binding domain and a calmodulin-binding site. Calmodulin specifically binds to the putative targeting site in a calcium-dependent manner. All *SlSRs *were highly yet differentially expressed during fruit development and ripening. Most notably, the expression of *SlSR2 *was scarcely detected at the mature green and breaker stages, two critical stages of fruit development and ripening; and *SlSR3L *and *SlSR4 *were expressed exclusively in fruit tissues. During the developmental span from 10 to 50 days post anthesis, the expression profiles of all seven *SlSR*s were dramatically altered in ripening mutant *rin *compared with wildtype fruit. By contrast, only minor alterations were noted for ripening mutant *nor *and *Nr *fruit. In addition, ethylene treatment of mature green wildtype fruit transiently stimulated expression of all *SlSR*s within one to two hours.

**Conclusions:**

This study indicates that *SlSR *expression is influenced by both the *Rin*-mediated developmental network and ethylene signaling. The results suggest that calcium signaling is involved in the regulation of fruit development and ripening through calcium/calmodulin/SlSR interactions.

## Background

Fleshy fruits, a significant part of the human diet, provide fiber, minerals and various nutraceuticals, and promote human health. Fruit quality and postharvest shelf life are dependent upon the control of ripening. Fruit ripening is a complex, genetically programmed process. Classically, fruits are grouped into two ripening types, climacteric and non-climacteric. The ripening of climacteric fruits such as tomato and apple, usually is triggered by biosynthesis of the gaseous hormone ethylene. In contrast, the ripening of non-climacteric fruits, such as strawberry and grape is mediated by an ethylene-independent process with little changes in respiration rate (reviewed in [1-3]). The fruit ripening process for both fruit types is affected by environmental cues such as temperature change, wounding and pathogen infection. Calcium has been shown to be important in controlling fruit ripening and quality by delaying ripening and maintaining firmness. Increasing the calcium concentration of fruit through preharvest sprays and postharvest calcium treatments, such as dipping and vacuum infiltration, maintains firmness and prevents decay in both climacteric and non-climacteric fruits [4-10]. Calcium is recognized as a critical element for rigidifying cell walls by cross-linking with pectins [11,12]. However, the molecular mechanisms whereby calcium retards fruit ripening remain elusive.

Signaling cascades mediating plant responses to environmental and hormonal cues often involve calcium as a second messenger (reviewed in [13-19]). Cellular calcium changes can be sensed and interpreted by calcium-binding proteins that function as signal sensors [20]. Calmodulin is one of the most well characterized calcium-sensors and functions as a modulator to other target proteins (reviewed in [21-23]). These proteins play roles in metabolism, ion transport, transcriptional regulation, protein phosphorylation, and other critical functions. Recently, a calcium/calmodulin-regulated SR/CAMTA transcription factor family has been shown to play an important role in the plant response to abiotic and biotic stresses. First identified in plants [24], *SR/CAMTA*s are present in all plant and animal species surveyed to date. In plants, *SR/CAMTAs *show differential responses to developmental signals and a variety of environmental signals. For instance, the tobacco ortholog *NtER1 *is an ethylene-responsive gene and highly expressed in senescing flowers and leaves [24]. Expression of the tomato ortholog *ER66 *is stimulated by ethylene, and is higher in fruit tissues at the red ripe stage than those at the mature green stage [25]. In *Arabidopsis*, six *AtSR*s differentially respond to a variety of external signals, such as cold, wounding and drought, as well as hormonal signals like ethylene and ABA [26-28]. The knockout of *AtSR1 *led to increased accumulation of salicylic acid and enhanced disease resistance [29]. Salicylic acid can reduce the expression of ACC synthase and affect ethylene biosynthesis [30,31]. *AtSR1/CAMTA3 *and *AtSR2/CAMTA1 *are also important for plant tolerance to low temperature [32]. Knockout of those genes significantly reduced cold tolerance. The genes affected by *AtSR1/CAMTA3 *include PR genes, expansin, beta-1,3-glucanase, phospholipase A2, accelerated cell death protein 6, and senescence associated gene 21 [33]. The *SR/CAMTA*s' primary target of CGCG *cis*-element has been suggested to be the major calcium-regulated *cis*-element [34] and the rapid wounding responsive element [35]. Therefore *SR/CAMTA*s may sit at the crossroads where calcium signaling intersects the ethylene, salicylic acid, wounding and cold signal transduction pathways. All of these signaling pathways have a major impact on fruit ripening and quality.

To investigate the role of calcium-regulated SR/CAMTA in fruit ripening, we selected tomato, a model fruit-bearing crop, because of its well characterized molecular basis of fruit ripening and its economic importance. Tomato (*Solanum lycopersicum*) is one of the most important horticultural crops. It has become an excellent model to study fleshy fruit development and ripening because highly developed genetic and molecular toolkits are available [3,36]. Single gene mutants representing ripening associated phenotypes have been well characterized (reviewed in [37]). These include the recessive ripening mutant *rin *(ripening-inhibitor), and dominant ripening mutants *Nor *(non-ripening), *Nr *(never-ripe), *Cnr *(colorless non-ripening) and *Gr *(green-ripe) [38-41]. Of them, the *Rin*, and *Nor *genes encode a MADS-box and a NAC-domain transcription factor, respectively, and act upstream of crucial ripening activities including ethylene production, suggesting that they regulate ethylene-independent ripening processes [39]. In contrast, *Nr *is directly involved in ethylene-dependent ripening because *Nr *encodes an ethylene receptor [38]. Here we report the isolation of seven *SlSR*s, the *SR/CAMTA *orthologs in tomato and characterization of their expression patterns during fruit development in wildtype fruits and ripening mutants, as well their response to ethylene treatment.

## Results

### Seven *SR/CAMTA*s are expressed in tomato

**Figure 1:**
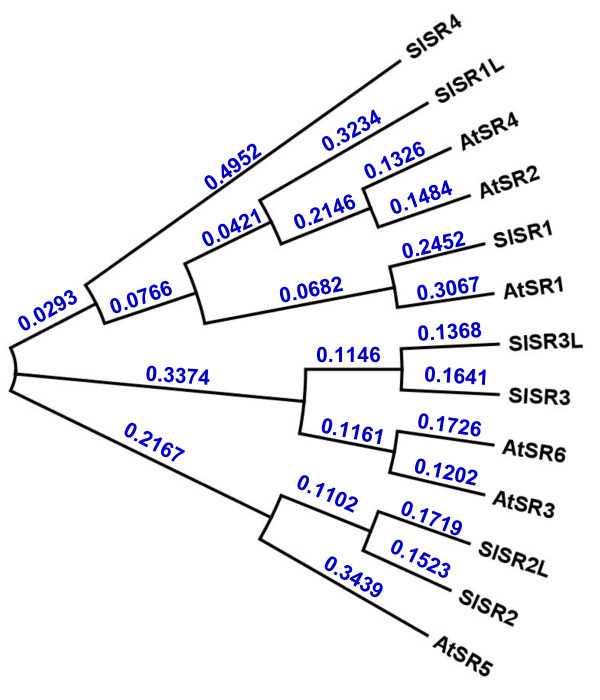
**Phylogenetic analysis of amino acid sequences encoded by seven tomato *SlSR*s and *SR/CAMTA *orthologs from *Arabidopsis thaliana***. The corresponding GenBank cDNA accession numbers or gene identification numbers are as follows: SlSR1, GU170838; SlSR1L, JN558810; SlSR2, JN566047; SlSR2L, JN566048; SlSR3, JN566049; SlSR3L, JN566051; SlSR4, JN566050; AtSR1, AT2G22300; AtSR2, At5G09410; AtSR3, At3G16940; AtSR4, At5G64220; AtSR5, At1G67310; AtSR6, At4G16150. Numbers at tree nodes indicate the genetic distances in substitutions per site.

There are six *SR/CAMTA*s (*AtSR1-6*) in the *Arabidopsis *genome and all of them are expressed in tissues [26]. To clone the tomato counterparts, the amino acid sequences of the six *Arabidopsis *SR/CAMTAs were used to search GenBank http://www.ncbi.nlm.nih.gov and tomato databases on the SOL Genomics Network http://solgenomics.net. There were about 50 EST clones and 16 Unigenes showing linear homology to *AtSR1-6*, but none of them included a full-length coding region. Analysis of the ESTs and Unigenes indicated that there are seven putative *SR/CAMTAs*, designated as *SlSR*s, expressed in tomato. To obtain the full length cDNAs, a mixture of total RNAs from various tissues and fruit pericarp at several developmental stages was used for reverse transcription and amplification with gene specific primers (Table 1). The primer sets for cloning three of them, *SlSR1, SlSR2 *and *SlSR3*, were designed directly from the available Unigenes/ESTs based on the amino acid sequence alignment with *Arabidopsis AtSRs *in the N-terminal and C-terminal regions. For the remaining four genes, their missing 5' and 3' ends were obtained using 5' and/or 3' RACE. The gene specific primer pairs were further utilized to amplify their full length coding regions. Three of the four *SlSR*s were named as *SlSR1L, SlSR2L *and *SlSR3L *because they showed high homology to *SlSR1, SlSR2 *and *SlSR3*, respectively. However, *SlSR4 *had no close relationship to any of the other six. All *SlSR *encoded polypeptides include the SR/CAMTA structural features: a conserved DNA-binding domain in the N-terminal portion, a calmodulin-binding domain in the C-terminal portion, and ankyrine repeats in the middle, suggesting that tomato *SlSR*s are true orthologs of the *SR/CAMTA *transcription factor family. As shown in Additional File 1, the deduced amino acid sequences of the seven *SlSR*s have 45-76% and 30-65% overall similarity and identity, respectively. However, there are over 80-93% similarity and 63-88% identity of amino acid sequences in the N-terminal DNA-binding domain and the C-terminal calmodulin-binding domain. The phylogenetic relationship of *SlSR *encoded polypeptides and *Arabidopsis *AtSRs was further analyzed (Figure 1). SlSR1 is closely related to AtSR1, SlSR2 and SlSR2L are in the same clade with AtSR5, and SlSR3 and SlSR3L are very similar to AtSR3 and AtSR6. In contrast, SlSR1L has a weak homology to AtSR2, AtSR4 and SlSR1, whereas SlSR4 is not closely related to any of them, suggesting that *SlSR4 *is a relatively new member of the tomato *SR/CAMTA *gene family.

**Table 1:** Primers Used for Gene Cloning and qPCR

SlSR1-P1*	ATGGCAGACAGTAGGCGTTA
SlSR1-P2*	TCAAGGTGCTGTAGGCATAAAA

SlSR1L-P1*	ATGGACATAACACAGATATTATCCG

SlSR1L-P2*	TTATTCAAATGCTATAGACATGAAAGTA

SlSR2-P1*	ATGGCAGAATCAGGATACAACACA

SlSR2-P2*	TTAGACATGTCCATGAGCAGTTG

SlSR2L-P1*	ATGGCGGAATCAGGATATGATATT

SlSR2L-P2*	CTAGATGGATGATTGACTGACCT

SlSR3-P1*	ATGGAAAGCAACAGAGCAGGAC

SlSR3-P2*	CTAGTTATCAGGATTGATAAGCCTT

SlSR3L-P1*	ATGGAAAGTAGCGTATCAGGACGA

SlSR3L-P2*	TTAGTCCATCTCAGTGTCAGGATTG

SlSR4-P1*	ATGGCAGTAGATCTTGAACAGATA

SlSR4-P2*	CTAAACTGGTGGTGATGACCTA

SlSR1-A**	CAGAAGGCTCTGGAAAGGGTAA

SlSR1-B**	AAATCAACTGCTTCCGCTGAGT

SlSR1L-A**	AAGTGAAGCAGGATGGTCCAAT

SlSR1L-B**	ATTGGCTAATCAAGGGGGAAAA

SlSR2-A**	CTTTGCACCCAGTTTTAAATGC

SlSR2-B**	GTAACAGCTCCAGCAAATGCAC

SlSR2L-A**	AAATGCTGAAGCCCTGAATTGT

SlSR2L-B**	ATTGGCTAATCAAGGGGGAAAA

SlSR3-A**	ACACAATAATGCTTCGCTGGAA

SlSR3-B**	GGGGCAATATCACAATGCTTTT

SlSR3L-A**	GCAATGGAAAGTAGCGTATCAGG

SlSR3L-B**	GGCAAGTTTACAGGCTTGACATT

SlSR4-A**	TTCTGGCCAAAATCCTCGTAAT

SlSR4-B**	TTGTGAAAATGCAGGCTCTTGT

E4-A**	ACCAGCAATATCTAGAGAAGGGTG

E4-B**	ATCATTGTCATGTTTATTCAAATTTAAAG

Actin-A**	GAAATAGCATAAGATGGCAGACG

Actin-B**	ATACCCACCATCACACCAGTAT

*Primers used for cloning; **Primers used for qPCR

### All *SlSR*s encode calcium/calmodulin-binding proteins

**Figure 2:**
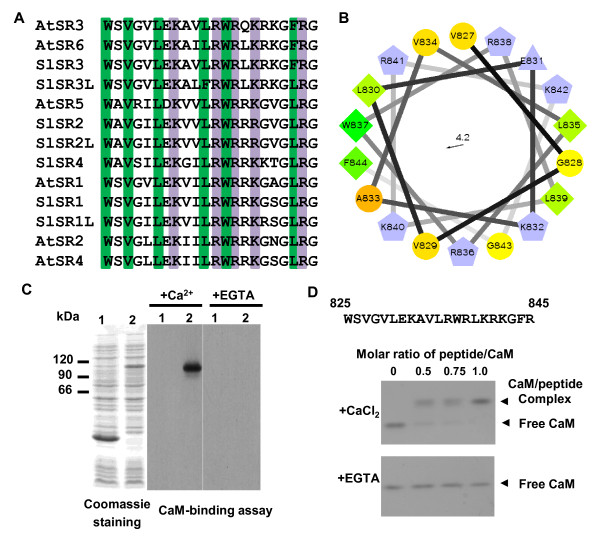
**SlSRs are calcium/calmodulin-binding proteins**. (**A**) Alignment of the conserved putative calmodulin-binding regions of SlSRs with AtSRs. The conserved amino acids are highlighted with either green (hydrophobic residues) or purple (positively charged residues). (**B**) Helical wheel projection showing the basic amphipathic α-helix structure in the predicted calcium/calmodulin-binding domain of SlSR3. (**C**) Calmodulin-binding overlay assay showing that calmodulin binds SlSR3 only in the presence of calcium. Total proteins from *E. coli *carrying pETSlSR3 were analyzed using SDS-PAGE, transferred onto a PVDF membrane and incubated with biotinylated calmodulin. 1, No IPTG induction; 2, IPTG added. (**D**) Gel mobility shift assay showing that calcium loaded calmodulin binds to the synthetic peptide corresponding to the putative calmodulin-binding region in SlSR3 (peptide amino acid sequence shown at the top of panel D). Arrows indicate the positions of free calmodulin (CaM) and the peptide-calmodulin complex.

All SR/CAMTA proteins reported thus far are known to be calcium/calmodulin-binding proteins. To ascertain whether tomato *SlSR*s encode calcium/calmodulin-binding proteins, their putative calmodulin-binding regions were aligned with the corresponding regions in *Arabidopsis *AtSRs (Figure 2A). The corresponding sites in AtSRs are well characterized calcium/calmodulin-bining regions and share very high homology with their counterparts in the SlSRs (overall > 90% amino acid sequence identity). Within this region, SlSR1, SlSR1L, SlSR2, SlSR2L and SlSR4 have almost the same amino acid sequence as AtSR1, and SlSR3 and SlSR3L show high similarity to AtSR3. All can form a basic amphipathic α-helix structure [42], which can be recognized by calmodulin. We selected SlSR3 as an example to show a basic amphipathic α-helix structure projection, where the hydrophobic half is on the left side, and the basic hydrophilic half is on the right side (Figure 2B). Furthermore, IPTG-induced bacterial expression of the full-length coding region of *SlSR3 *fused with a His_6_-tag yielded a recombinant SlSR3 band with a molecular mass of ~105 kDa, as determined by SDS-PAGE (Figure 2C). A calmodulin overlay assay showed that calmodulin binds SlSR3 in the presence of calcium, but not in the presence of EGTA, a calcium chelator. Finally, a gel-mobility shift assay was used to confirm calmodulin binding specifically to the putative calmodulin-binding site in SlSR3 (Figure 2D). A synthesized peptide corresponding to the 21-amino acid calmodulin-binding region of SlSR3 was incubated with calmodulin in the presence of calcium or EGTA. After separation by native PAGE, the calmodulin-peptide complex was observed in the presence of calcium. The intensity of the complex band increased following an increase in the peptide-calmodulin ratio. By contrast, only a free calmodulin band appeared in the presence of EGTA. To investigate whether all seven SlSRs are calcium/calmodulin-binding proteins, three additional peptides corresponding to the putative calmodulin-binding regions in SlSR1, SlSR2/SlSR2L, and SlSR4 were synthesized and assayed. The gel mobility shift assays showed that calmodulin binds to each of the three peptides only in the presence of calcium (Additional File 2). Since the corresponding region of SlSR1 and SlSR3L to SlSR1L and SlSR3, respectively contains just one or two conservative amino acid substitution(s), the results indicate that all SlSRs are calcium/calmodulin-binding proteins.

### *SlSR*s are differentially expressed in tomato tissues and during fruit development

**Figure 3:**
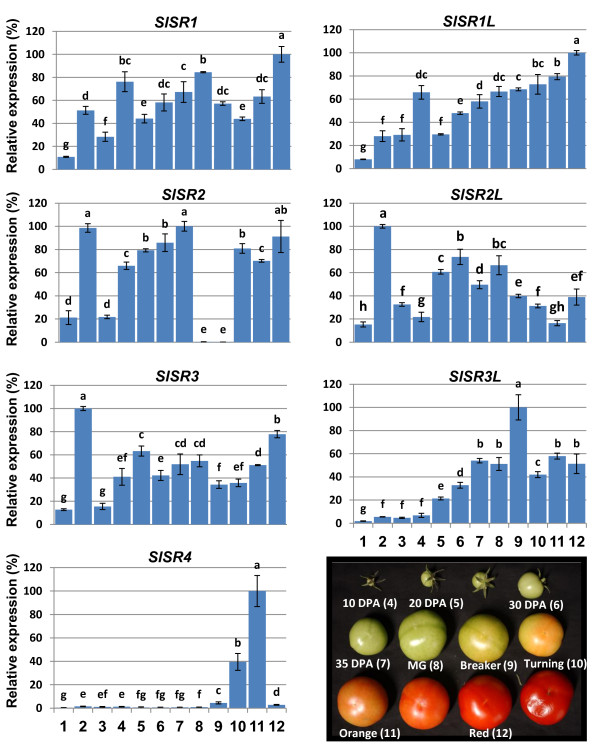
**Expression patterns of *SlSR *genes in different tissues and during fruit development**. Transcription levels of *SlSR*s were investigated by RT-qPCR. Relative gene expression levels (highest value = 100%) are shown after normalization with actin transcript values. Error bars represent standard error of the mean. For each gene, different letters indicate significant differences among mean values (*P *< 0.05; *t*-test). Numbers on the x-axis represent the following: two-week old seedling leaves (1) and roots (2), flowers (3), fruit at 10, 20, 30, and 35 DPA (4-7), and fruit at the mature green, breaker, turning, pink and red stages (8-12). The RT-qPCR analyses were repeated at least three times in three independent experiments. Photos of fruit at the nine developmental stages assessed (4-12; 10 DPA to red ripe) are shown on the bottom right.

We cloned seven *SlSR*s from the pooled cDNA derived from an array of tomato tissues, indicating that there are seven expressed tomato *SR/CAMTA*s. To investigate the temporal and spatial expression profiles of these *SlSR*s, the relative levels of their transcripts in various tissues and in fruit at different developmental stages were quantified using RT-qPCR. All expression levels as related to the expression of *actin *gene are shown in Figure 3. Overall, the expression levels for all *SlSR*s were low in leaves and flowers, and relatively high in fruit. Some genes, such as *SlSR2, SlSR2L *and *SlSR3*, were highly expressed in roots. All the *SlSR*s exhibited differential expression patterns in various tissues and at various fruit development stages.

During fruit development and ripening, *SlSR1 *showed three peaks of expression, which appeared at 10 DPA, the mature green stage and the red ripe stage. *SlSR1L *transcript increased gradually after 25 DPA during fruit enlargement and ripening, while *SlSR2L *had a bell curve expression pattern, with the peak around the mature green stage. *SlSR3 *expression levels exhibited the least variation among different stages (less than two fold difference) with the highest expression at the red ripe stage. Expression of *SlSR3L *and *SlSR4 *was shown to be fruit specific. Moreover, *SlSR4 *was expressed only in turning and orange fruit, while *SlSR3L *transcript levels peaked at the breaker stage. The most interesting expression pattern was that of *SlSR2*. It was highly expressed in fruit at all developmental stages except mature green and breaker, the earliest stages of ripening, when expression was undetectable. To exclude the possibility that the unique *SlSR2 *expression pattern is unique to the Rutgers cultivar, we examined fruit of the cultivars Moneymaker and Alisa Craig as well, and found that *SlSR2 *transcript was scarcely detectable at the mature green stage, too (data not shown). Overall, these results indicate that the seven *SlSR*s are differentially expressed during fruit development and ripening.

### Expression of *SlSR*s in fruit of tomato ripening mutants

**Figure 4:**
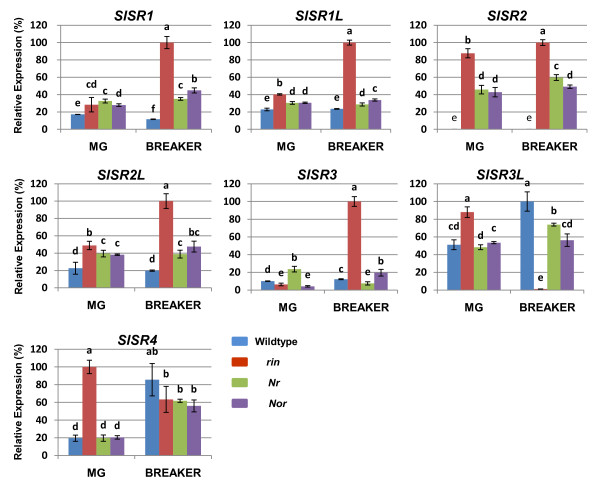
**Expression patterns of *SlSR*s in tomato ripening mutant fruit**. Total RNA from the *rin, Nor *and *Nr *fruit at mature green (MG) and breaker equivalent stages was subjected to RT-qPCR analysis. Relative gene expression levels (highest value = 100%) are shown following normalization with actin transcript values. Error bars represent standard error of the mean. For each gene, different letters indicate significant differences among mean values (*P *value < 0.05; *t*-test). The analyses were repeated at least three times in three independent experiments.

In the earliest stages of ripening, tomato fruit transition from mature green, when maximum size is attained but the skin and flesh remain green, to breaker, when color change and other aspects of ripening are first evident. Several tomato spontaneous ripening mutants, including *rin, Nor *and *Nr*, have normal fruit size but fail to ripen. To determine whether *SlSR*s have any relationship to the defined fruit ripening genes that are deficient in these mutants, we compared expression levels of the seven *SlSR*s in wildtype fruit at the mature green and breaker stages with those in mutant fruit at the chronologically equivalent stages 45 and 50 DPA, respectively (Figure 4).

At the mature green stage/45 DPA, expression levels of *SlSR1, SlSR1L *and *SlSR2L *were consistently higher in the mutant compared with wildtype fruit. Specifically, *SlSR1L *and *SlSR2L *expression was about twofold higher in *rin*, and about 50% higher in *Nor *and *Nr*, whereas for *SlSR1 *transcript was roughly 50% more abundant in all three mutants. For both *SlSR3L *and *SlSR4*, increased expression relative to wildtype was noted only in the *rin *mutant. In contrast, expression of *SlSR3 *was lower in *rin *and *Nor*, while in *Nr *it was twofold higher than in wildtype fruit. The most dramatic changes were found for *SlSR2 *and *SlSR4*. *SlSR2 *was not expressed in wildtype fruit, but was highly expressed in *rin*, as well as in *Nr *and *Nor *(about 40% relative to *rin*). *SlSR4 *expression was fivefold higher in *rin *compared with wildtype fruit.

At the breaker stage/50 DPA, the most obvious trend was that, except for *SlSR4*, the expression levels of all *SlSR*s was significantly altered in *rin *compared with wildtype fruit (Figure 4). *SlSR3L *was completely suppressed in *rin *fruit, whereas expression levels of the other five genes were at least fivefold higher than in wildtype. In contrast, with the exception of *SlSR2*, differences in *SlSR *expression levels relative to those in wildtype fruit were much less pronounced in *Nor *and *Nr *fruit. Similar to the expression profiles at the mature green stage, *SlSR2 *transcript was not detectable in wildtype fruit, but was abundant in fruit of all three mutants, with the highest level in *rin*. In contrast, *SlSR4 *expression was not significantly different in fruit of wildtype and the three ripening mutants. Thus, overall, the *rin *mutation had by far the most marked influence on *SlSR *gene expression at the mature green and breaker equivalent stages of fruit development.

**Figure 5:**
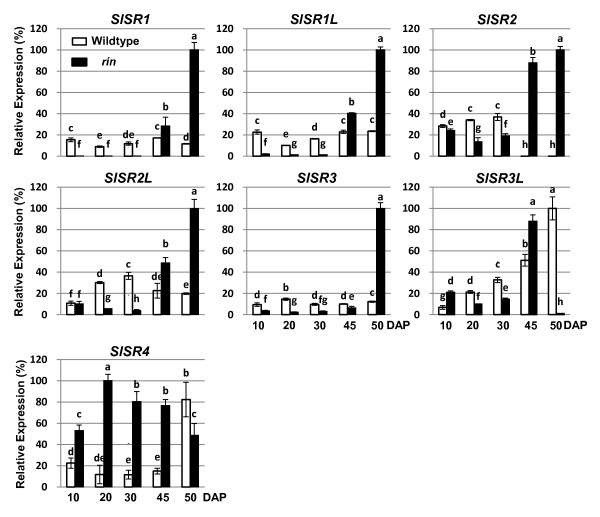
**Expression patterns of *SlSR*s in *rin *fruit during development**. Total RNA from the *rin *and wildtype fruit at 10, 20, 30, 45 and 50 DPA was subjected to RT-qPCR analysis. Relative gene expression levels (highest value = 100%) are shown following normalization with actin transcript values. Error bars represent standard error of the mean. For each gene, different letters indicate significant differences among mean values (*P *< 0.05; *t*-test). The analyses were repeated at least three times in three independent experiments.

We further investigated whether expression of *SlSR *genes differs in *rin *and wildtype fruit prior to the mature green stage, comparing *SlSR *mRNA levels at 10, 20 and 30 DPA (Figure 5). All the *SlSR*s except *SlSR4 *had lower expression in *rin *fruit before 45 DPA, which is equivalent to the mature green stage in wildtype fruit. By contrast, *SlSR4 *showed significantly higher expression in *rin *than in wildtype fruit at 10, 20, 30, and 45 DPA, and then declined below the wildtype level at 50 DPA (equivalent to breaker stage), in part due to a fivefold increase in *SlSR4 *transcript in wildtype. Thus, *SlSR *expression patterns are altered in *rin *mutant fruit during development, with dramatic changes relative to the patterns in wildtype fruit occurring at the mature green and breaker equivalent stages. These differences in *SlSR *expression are most likely related to fruit ripening rather than expansion growth since *rin *yields normal sized fruit.

### *SlSR*s are early ethylene responsive genes

**Figure 6:**
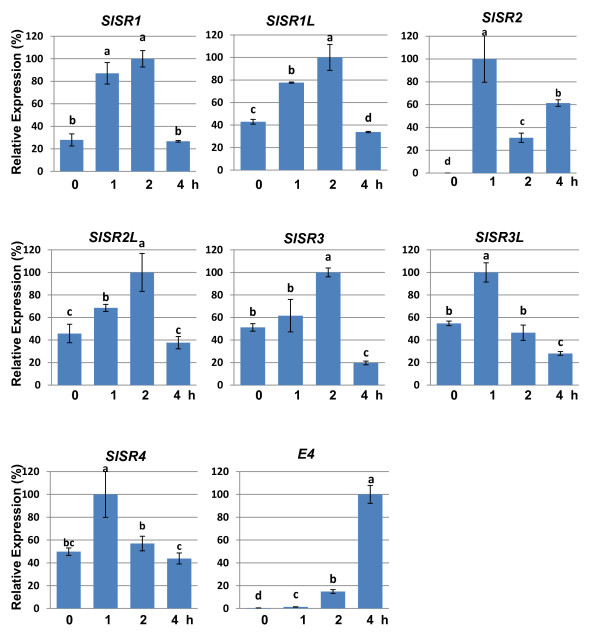
***SlSRs *expression is transiently stimulated by ethylene treatment**. Transcription levels of *SlSR *genes were investigated by RT-qPCR. Tomato fruits (cv. Rutgers) at the mature green stage were treated with 100 ppm ethylene for 0, 1, 2, or 4 h. Relative gene expression levels (highest value = 100%) are shown following normalization with actin transcript values. E4, a well characterized ethylene responsive gene, was used as a positive control. Error bars represent standard error of the mean. For each gene, different letters indicate significant differences among mean values (*P *< 0.05; *t*-test). The analyses were repeated at least three times in three independent experiments.

Ethylene is the major phytohormone controlling tomato fruit ripening and senescence. In tobacco and *Arabidopsis, SR/CAMTA*s are responsive to ethylene treatment [24,26,28]. To determine if all *SlSR *genes in tomato fruit are responsive to ethylene, we selected mature green stage fruit for ethylene treatment because little endogenous ethylene is produced at this stage. As shown in Figure 6, expression of all *SlSR*s was transiently up-regulated by exogenous ethylene treatment. The ethylene stimulation or induction for all the genes was very rapid, usually within one hour after treatment. In particular, *SlSR2*, which is not expressed at the mature green stage, was induced by 100 ppm ethylene within one hour after treatment. *SlSR1 *was stimulated about fourfold by ethylene and peaked two hours after ethylene treatment. The remaining genes were stimulated about twofold by ethylene within one hour and peaked at one or two hours after treatment. For each of the *SlSR*s with the exception of *SlSR2*, the stimulation of expression by ethylene was transient, i.e. after four hours transcript levels were the same or lower than those at zero hours. By contrast, *SlSR2 *transcript declined about threefold from one to two hours but then increased two fold from two to four hours. *E4*, a well characterized ethylene responsive gene [43], was induced dramatically by ethylene, indicating that the ethylene treatment was effective. These results suggest that *SlSR*s are early ethylene responsive genes.

## Discussion

In this study we isolated seven *SR/CAMTA *orthologs from tomato, designated as *SlSR*s, which were all shown to be expressed in fruit pericarp tissue. Analyses of *SlSR *expression patterns during fruit development and ripening demonstrated that the expression levels of these genes are developmentally regulated. In addition, it was shown that their expression is stimulated by ethylene. Fruit ripening is a complex developmental process, and in climacteric fruits such as tomato, ripening is controlled by a combination of developmental signals and the gaseous hormone ethylene. Characterization of tomato spontaneous ripening mutants has helped to elucidate control elements and regulatory pathways involved in climacteric ripening in this useful model fruit. *Nr *was the first cloned ripening gene and was shown to encode an ethylene receptor [38]. The mutation of *Nr *affects the ethylene perception and hence blocks fruit ripening. *Gr *is another gene associated with ethylene signalling [41]. On other hand, *Rin, Nor *and *Cnr *were were all found to encode transcription factors or a protein regulating transcription [39,40]. The *rin *mutation is especially interesting since it has been used to breed many commercial tomato hybrids which exhibit slow fruit ripening and long shelf life. *RIN *codes for a MADS-box protein of the SEPALLATA clade [39]. Homologues of *RIN *have been found to be expressed in grape [44] and strawberry [45] and others, thus suggesting a possible involvement in fruit development and ripening in both climacteric and non-climacteric fruits. In tomato, both *rin *and *Nor *fruits are responsive to ethylene, as shown by monitoring expression of ethylene-inducible genes. However, neither mutant ripens after ethylene treatment, suggesting that both genes are located upstream of crucial ripening activities (including ethylene production) and function in an ethylene-independent manner [3,39]. It is still unclear what the hierarchy is between these transcription networks and how the ethylene-dependent and ethylene-independent pathways coordinate to control the ripening process. Our results show that the expression patterns of *SlSR*s are altered in *rin, Nor *and *Nr *mutants compared with wildtype fruit, and the most significant changes occur in *rin *fruit. This raises the possibility that *SlSR*s are downstream targets of *RIN*. However, since ethylene treatment can stimulate *SlSR *expression, *SlSR*s could be regulated by both the ethylene-independent developmental network and ethylene-mediated signaling.

Calcium is recognized as a universal second messenger, which mediates responses to external stimuli and hormonal changes in plants via calcium sensors such as calmodulin [14,16,18-23]. Calcium treatment has been used to delay fruit ripening and senescence, and to maintain fruit quality [5-7,9,46]. Like all other *SR/CAMTA*s that have been characterized, the seven *SlSR*s encode a type of calcium/calmodulin-binding transcription factor. It has been reported that calcium/calmodulin-binding to *SR/CAMTA*s in *Arabidopsis *and *Drosophila *is crucial for their *in vivo *functions [29,47,48]. Therefore, it can reasonably be speculated that calcium regulates fruit development and ripening by forming a calcium/calmodulin complex to activate SlSRs, which then modulate the expression of down-stream genes. Possibly, SlSRs serve as a coordinator for multiple signaling pathways involved in the regulation of fruit development and ripening. Similar to *RIN, SR/CAMTA *is present in both climacteric and non-climacteric fruits, and therefore could function during non-climacteric fruit development and ripening as well.

Based on their expression patterns, *SlSR*s share some similarities yet exhibit substantial differences as well. Similarities include their positive responsiveness to ethylene treatment and their dramatically altered expression levels in *rin *mutant compared with wildtype fruit. However, the seven *SlSR*s exhibit different expression patterns during fruit development and ripening, suggesting that while they have some redundancy, individual genes may have specific functions. Interestingly, expression of *SlSR3L*, a fruit specific gene, is suppressed at the breaker equivalent stage (50 DPA) in *rin *mutant fruit. Conversely, other *SlSR*s, except *SlSR4*, show significantly increased expression at the breaker stage in *rin *compared with wildtype fruit, suggesting that *SlSR *expression is regulated positively and negatively by the *RIN*-mediated fruit ripening network. It is noteworthy that *SlSR2 *exhibited a uniquely different expression pattern in developing fruit when compared with the other six *SlSR*s, i.e. high expression during fruit enlargement, complete suppression during the mature green and breaker stages, and rapid induction with ripening after breaker. A similar expression pattern was reported for the tomato β-galactosidase gene *TBG5 *[49]. β-Galactosidase is important for hemicellulosic modifications that occur during cell division, cell growth and fruit ripening. It will be interesting to investigate whether there is any relationship between *SlSR2 *and cell wall modification.

A prior study showed that SR/CAMTAs specifically recognize the CGCG-box *cis*-element [26]. For example, AtSR1, an *Arabidopsis *SR/CAMTA ortholog, targets the CGCG-box in the promoter of *EDS1*, a key gene for the biosynthesis of salicylic acid [29]. Salicylic acid has been shown to down-regulate the expression of ACC synthase and thereby reduce ethylene biosynthesis [30,31], and to also delay fruit ripening [50,51]. Based on microarray assays, other possible targets identified for *AtSR1 *include genes encoding expansin, β-1,3-glucanase, phospholipase A2, accelerated cell death protein 6, and senescence associated protein 21 [33]. Assuming that SlSRs similarly regulate gene expression via binding to CGCG-box *cis*-elements, it is possible to identify SlSRs targets by searching those tomato genes carrying CGCG-box(s) in their promoter regions.

## Conclusions

We have isolated seven *SlSR *genes, the tomato *SR/CAMTA *orthologs. The expression levels of *SlSR*s are differentially regulated mainly by development signals, as well as by ethylene. Importantly, *SlSR *expression patterns during fruit development are altered in *rin*, a tomato ripening mutant, suggesting that *SlSR*s are located downstream of the *Rin*-regulated network. *SlSR*s encode calcium/camodulin-regulated transcription factors. Our data suggest that *SlSR*s act as a signal node candidate connecting developmental, ethylene-mediated and calcium-mediated signals, thereby regulating fruit development and ripening. Further functional studies of *SlSR*s will help to elucidate the signaling networks involved in these processes.

## Methods

### Plant materials and treatments

Tomato plants (*Solanum lycopersicum *cv Rutgers) were grown in a greenhouse at 28°C with a 16 h/8 h (light/dark) photoperiod. The ripening mutants *rin, Nor *and *Nr *were all in the cv Rutgers background. Flowers were tagged at anthesis and manually pollinated, and fruit were harvested according to the number of DPA (days post anthesis) or based on their surface color using ripening stages set by USDA. The equivalent stages to mature green and breaker stages for three ripening mutants are 45 and 50 DPA, respectively [49].

Fruit used in ethylene treatment experiments were harvested at the mature green stage and incubated under ambient conditions overnight to reduce harvest shock. Thereafter the fruit were sealed in a jar with 100 ppm ethylene for different time periods. Pericarp tissue excised from the fruit was frozen in liquid nitrogen and stored in -80°C.

### Cloning of tomato *SR/CAMTA *genes

Six *Arabidopsis SR/CAMTA *orthologs were used to search tomato EST clone collections and Unigene database http://solgenomics.net/tools/blast/index.pl. The primers used for cloning *SlSR*s were designed from the 5' ends and 3' ends of putative coding regions according to the linear alignment to *Arabidopsis *genes. For those cDNA fragments lacking 5' and/or 3' ends of the coding regions, 5'-RACE and/or 3'-RACE were performed using the 5' and/or 3' RACE kit (Invitrogen, USA) to obtain the missing regions following the manufacturer's instruction. *SlSR*s were amplified by PCR using *Pfx *DNA polymerase (Invitrogen, USA) from the mix of cDNAs from different tomato tissues and fruit at different stages described below using gene specific primers (Table 1). The PCR fragments were cloned in a TA cloning kit (Invitrogen, USA) after adding adenine and sequenced from both ends.

### RNA extraction and RT-qPCR

Total RNA was isolated from frozen tissue using the RNeasy Plant Mini Kit following the manufacturer's instructions (Qiagen, USA). After DNase digestion, the absorbance at 260 nm was measured using a nanodrop spectrophotometer to ensure equal amount of RNA used in the cDNA synthesis reactions among samples. One μg of total RNA was used to synthesize cDNA with the oligo-(dT)_18 _primer following the instructions of the Superscript III kit (Invitrogen, USA). Quantitative Real-Time PCR (RT-qPCR) analysis of cDNA was performed on a CFX96 Real-Time System (Bio-RAD, USA) using SYBR Green detection chemistry. Gene specific primers were designed with Primer3 software http://frodo.wi.mit.edu/primer3/. These primers are listed in Table 1. The efficiency coefficient E was calculated for all primer pairs individually by plotting the relationship between Ct value (threshold cycle) and log[cDNA]. The following thermal cycle conditions were used: 95°C for 2 min, followed by 45 cycles of 95°C for 5 s and 60°C for 20 s. All reactions were performed in triplicate from three independent samples. Following PCR, a melting curve analysis was performed. Relative quantification of specific mRNA levels were analyzed using the cycle threshold (Ct) 2^ΔΔCt ^method. Relative expression levels are normalized using the housekeeping gene actin (accession number: X55749) and shown in percentage (highest value = 100%). Student's *t *test (P_0.05_) was used to determine the significant difference of relative expression of individual genes among different tissues and fruit developmental stages, or wildtype and mutant fruit or ethylene treatments (Microsoft Excel 2007).

### Phylogenetic analysis

Multiple sequence alignments of the encoded full-length SR/CAMTA proteins were assembled with the Geneious software package http://www.geneious.com/. Phylogenetic reconstruction was obtained by the neighboring-joining method. Genetic distances were calculated as substitutions per site using the Jukes-Cantor model.

### Calmodulin overlay assay

The template coding for SlSR3 were produced by PCR amplification from the cDNA with *SlSR3-*specific oligonucleotides containing appropriate restriction sites (*Nde*I at the 5'-end and *Bam*HI at the 3'-end) for cloning into the downstream of the His_6 _tag in a pET-30b expression vector (Novagen, USA). The nucleotide sequence of the cloned fragment produced by PCR amplification was determined after cloning into the pET-30b vector, sequencing from both sides of the pET-30b cloning sites. The recombinant SlSR3 was expressed in *Escherichia coli *strain BL21(DE3) pLysS. Proteins were separated by SDS-polyacrylamide gel electrophoresis (SDS-PAGE), electrotransferred onto a polyvinylidene difluoride membrane (Millipore), and treated with a biotinylated calmodulin with 1 mM CaCl_2 _or 2 mM EGTA as previously described [24].

### Peptide binding to calmodulin

The synthetic peptides were prepared in Genemed Synthesis Inc. Samples containing 240 pmol (4 μg) of bovine calmodulin (Sigma) and different amounts of purified synthetic peptides in 100 mm Tris-HCl (pH 7.2) and either 1 mM CaCl_2 _or 2 mM EGTA in a total volume of 30 μl were incubated for 1 h at room temperature. The samples were analyzed by nondenaturing PAGE as described [24].

### *SlSRs *accession numbers

Accession numbers of tomato *SlSR *genes deposited in GenBank: *SlSR1*, GU170838; *SlSR1L*, JN558810; *SlSR2*, JN566047; *SlSR2L*, JN566048; *SlSR3*, JN566049; *SlSR3L*, JN566051; *SlSR4*, JN566050.

## Abbreviations

CaM: calmodulin; DPA: days post anthesis; EGTA: ethylene glycol-bis(2-aminoethylether)-*N, N, N', N'*-tetraacetic acid; IPTG: isopropyl β-D-1-thiogalactopyranoside; *Nor*, tomato non-ripening mutant; *Nr*, tomato never-ripe mutant; RACE, rapid amplification of cDNA ends; *rin*, tomato ripening inhibitor mutant; SDS-PAGE: sodium dodecyl sulfate polyacrylamide gel electrophoresis.

## Authors' contributions

TY conceived the study, designed all the experiments and performed *in silicon *and biochemical analyses. HP carried out all the gene expression studies and statistical analysis. TY, BDW and WSC interpreted the experimental dada and participated in writing the manuscript. All the authors read and approved the final manuscript.

## Supplementary Material

Additional file 1 **Amino acid sequence alignment of SlSRs with *Arabidopsis *orthologs**. The conserved DNA-binding region near the N-terminus, ankyrin repeats in the middle and calcium/calmodulin-binding site near the C-terminus are underlined. The corresponding GenBank cDNA accession numbers or gene identification numbers are as follows: SlSR1, GU170838; SlSR1L, JN558810; SlSR2, JN566047; SlSR2L, JN566048; SlSR3, JN566049; SlSR3L, JN566051; SlSR4, JN566050; AtSR1, AT2G22300; AtSR2, At5G09410; AtSR3, At3G16940; AtSR4, At5G64220; AtSR5, At1G67310; AtSR6, At4G16150. Click here for file

Additional file 2 **Calcium/calmodulin binding to peptides from SlSR1, SlSR2/SlSR2L, and SlSR4**. Three peptides corresponding to the putative calmodulin-binding domains of SlSR1, SlSR2/SlSR2L, and SlSR4 were synthesized. Gel mobility shift assay showing that calcium-loaded calmodulin binds to all three peptides in the presence of calcium. Arrows indicate the positions of free calmodulin (CaM) and the peptide-calmodulin complexes. Click here for file
