# Supplementary material for: Characterization of a calcium/calmodulin-regulated SR/CAMTA gene family during tomato fruit development and ripening
Source: BMC Plant Biol. 2012 Feb 13;12:19. doi: 10.1186/1471-2229-12-19 (PMC3292969; doi:10.1186/1471-2229-12-19)
Supplement: Additional file 1 — Amino acid sequence alignment of SlSRs with Arabidopsis orthologs. The conserved DNA-binding region near the N-terminus, ankyrin repeats in the middle and calcium/calmodulin-binding site near the C-terminus are underlined. The corresponding GenBank cDNA accession numbers or gene identification numbers are as follows: SlSR1, GU170838; SlSR1L, JN558810; SlSR2, JN566047; SlSR2L, JN566048; SlSR3, JN566049; SlSR3L, JN566051; SlSR4, JN566050; AtSR1, AT2G22300; AtSR2, At5G09410; AtSR3, At3G16940; AtSR4, At5G64220; AtSR5, At1G67310; AtSR6, At4G16150. [file 1471-2229-12-19-S1.PDF]

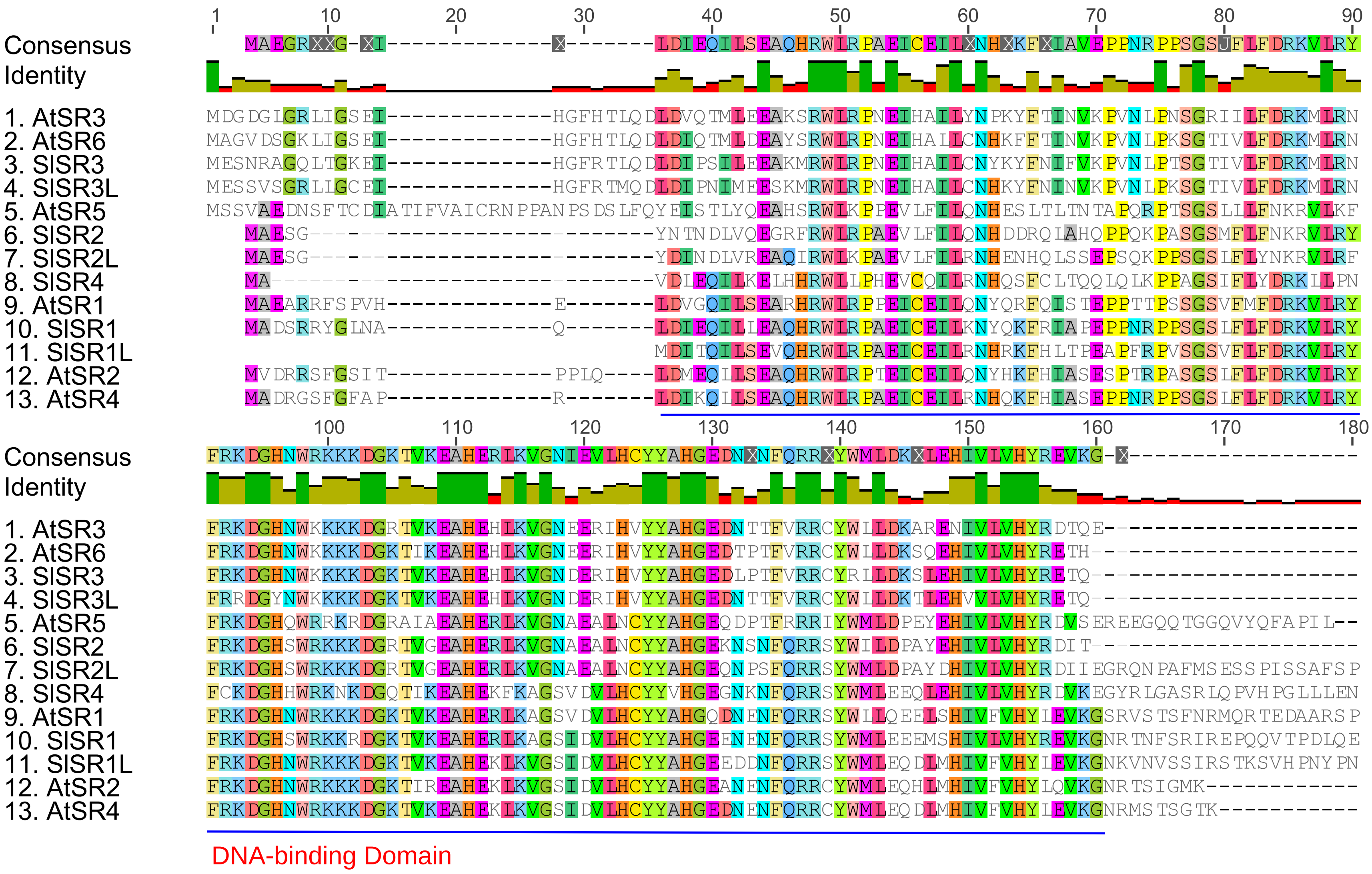

Additional File 1

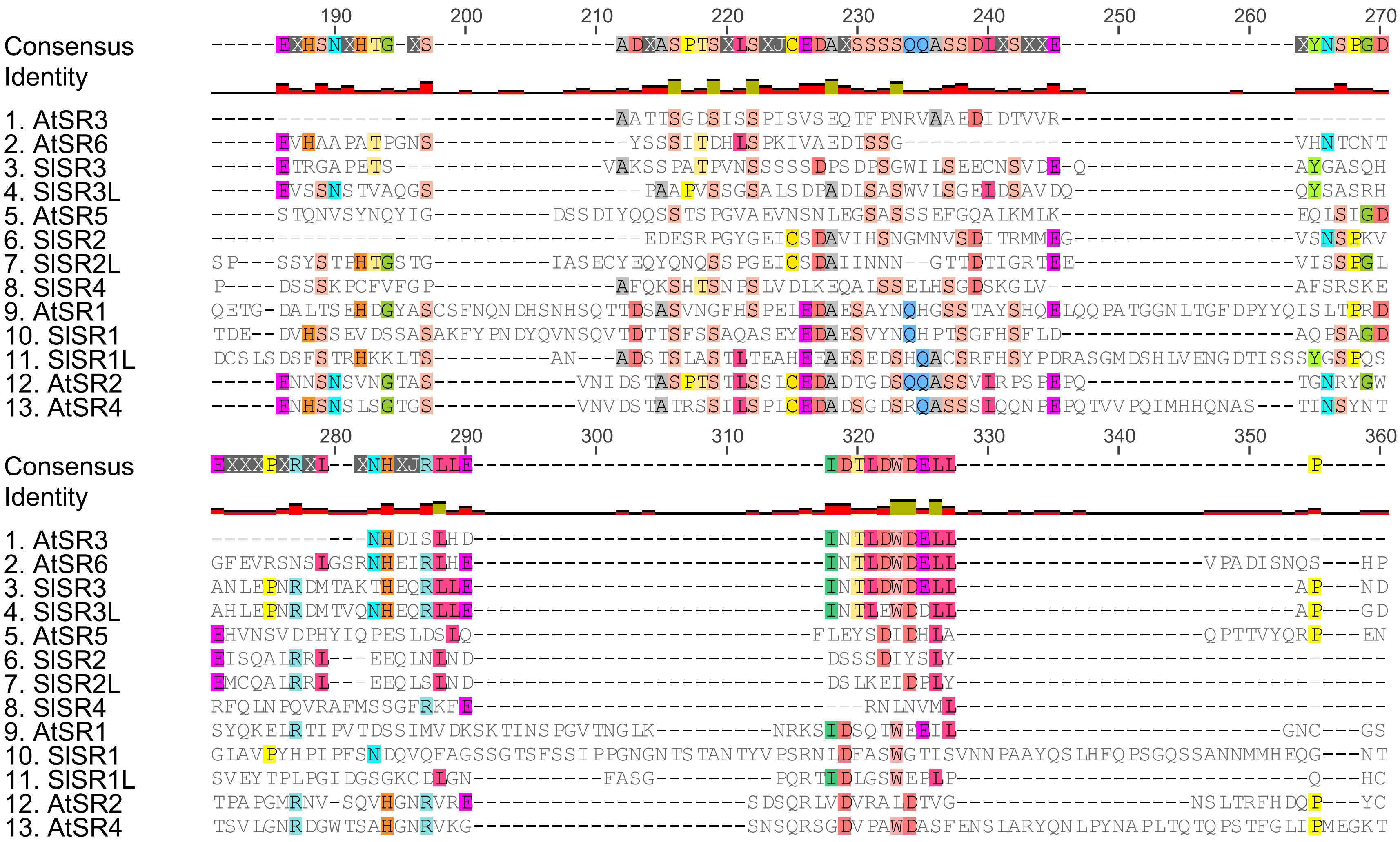
190
200
210
220
230
240
250
260
270
Consensus
Identity
1. AtSR3
2. AtSR6
3. SlSR3
4. SlSR3L
5. AtSR5
6. SlSR2
7. SlSR2L
8. SlSR4
9. AtSR1
10. SlSR1
11. SlSR1L
12. AtSR2
13. AtSR4
280
290
300
310
320
330
340
350
360
Consensus
Identity
1. AtSR3
2. AtSR6
3. SlSR3
4. SlSR3L
5. AtSR5
6. SlSR2
7. SlSR2L
8. SlSR4
9. AtSR1
10. SlSR1
11. SlSR1L
12. AtSR2
13. AtSR4

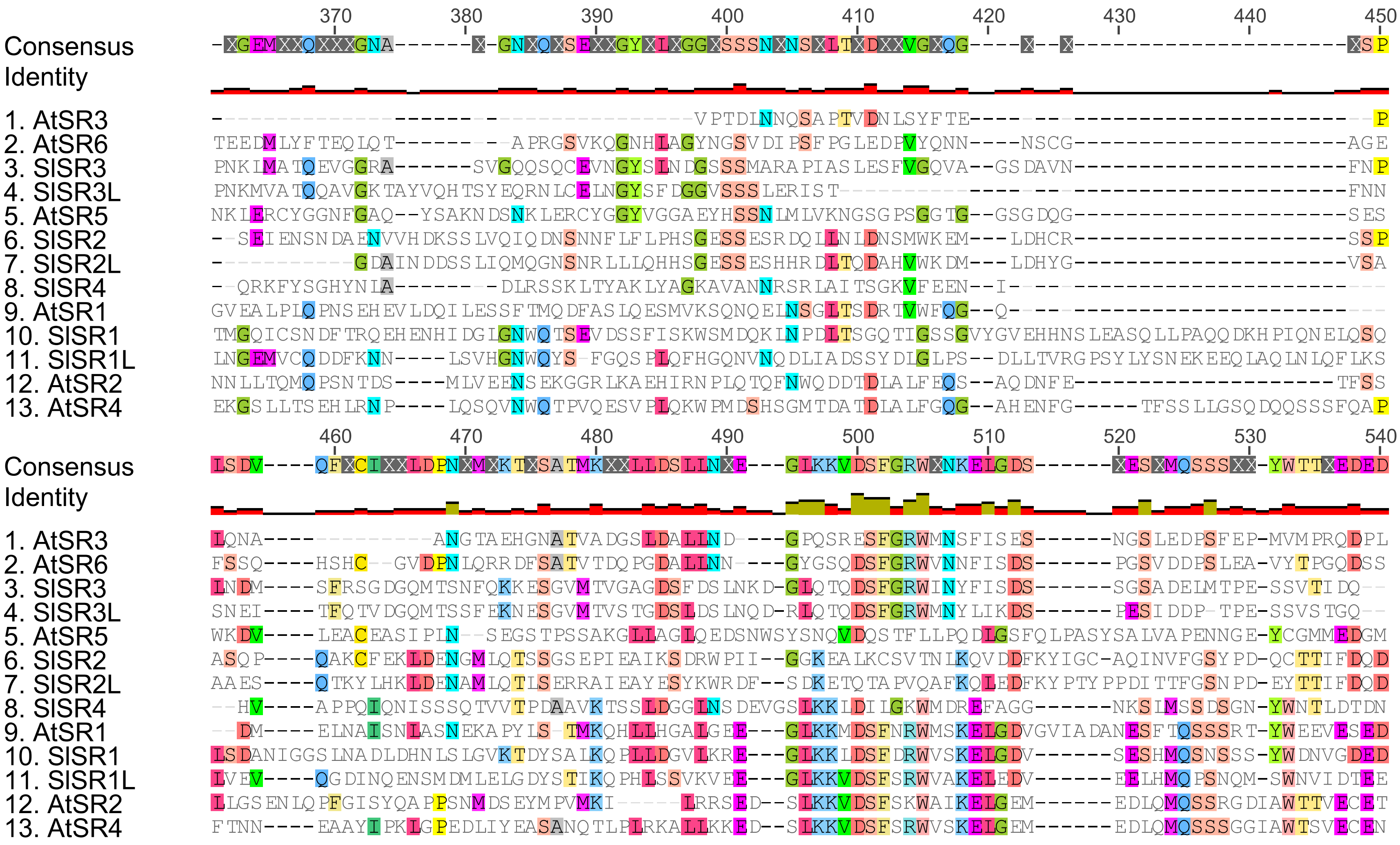

Consensus
Identity
1. AtSR3
2. AtSR6
3. SlSR3
4. SlSR3L
5. AtSR5
6. SlSR2
7. SlSR2L
8. SlSR4
9. AtSR1
10. SlSR1
11. SlSR1L
12. AtSR2
13. AtSR4

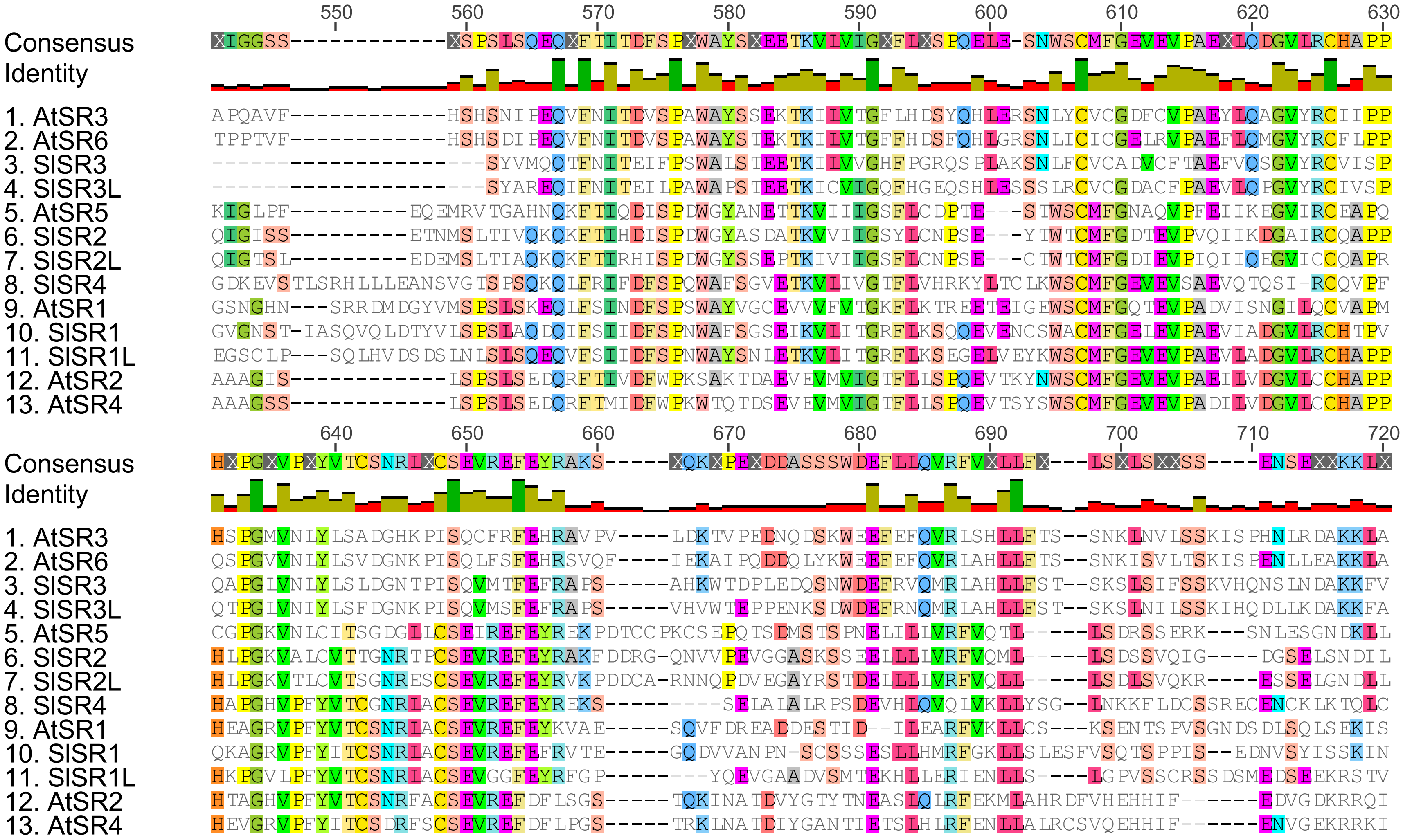

550
560
570
580
590
600
610
620
630
Consensus
Identity
1. AtSR3
2. AtSR6
3. SlSR3
4. SlSR3L
5. AtSR5
6. SlSR2
7. SlSR2L
8. SlSR4
9. AtSR1
10. SlSR1
11. SlSR1L
12. AtSR2
13. AtSR4
640
650
660
670
680
690
700
710
720
Consensus
Identity
1. AtSR3
2. AtSR6
3. SlSR3
4. SlSR3L
5. AtSR5
6. SlSR2
7. SlSR2L
8. SlSR4
9. AtSR1
10. SlSR1
11. SlSR1L
12. AtSR2
13. AtSR4

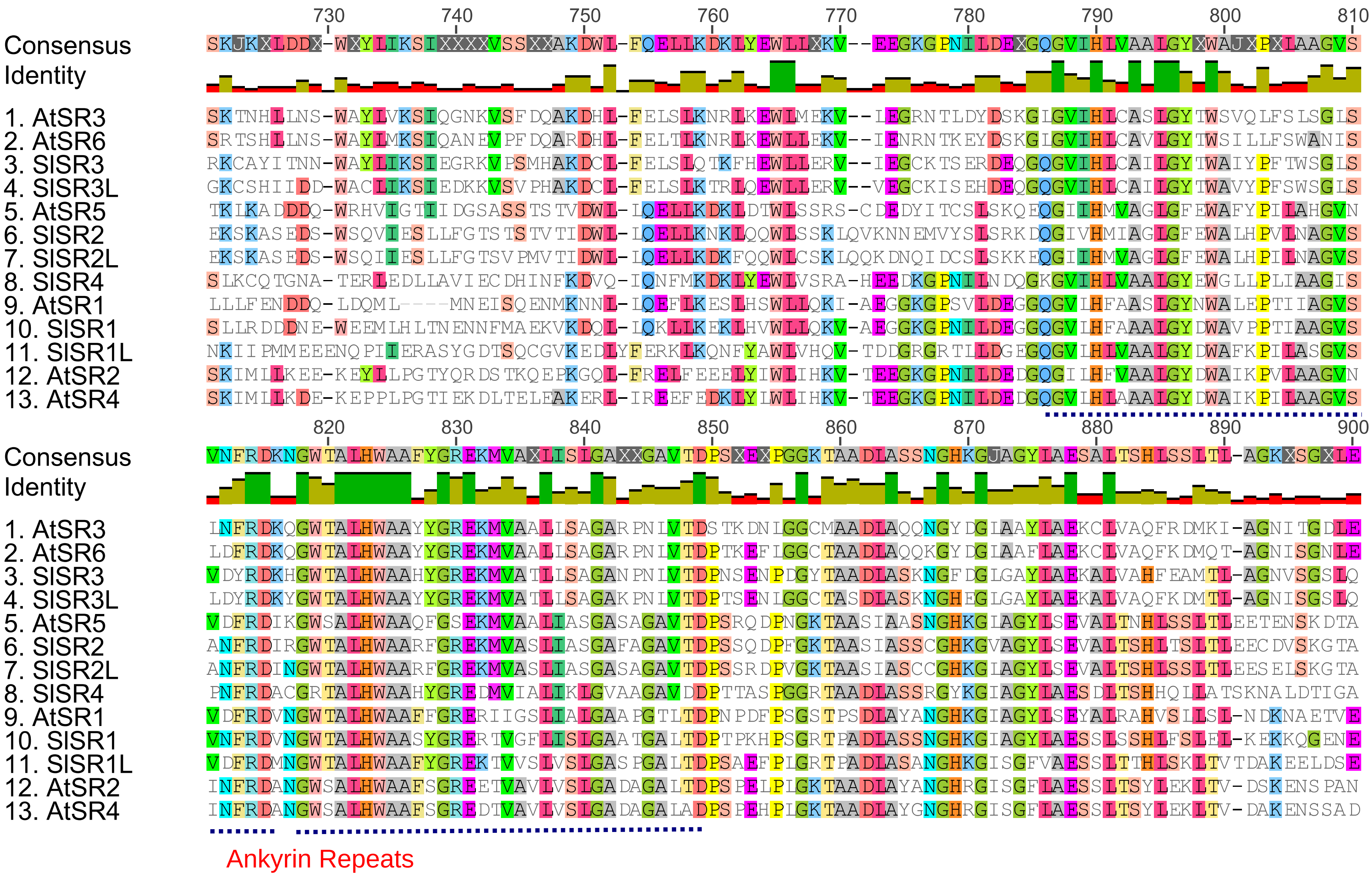

730
740
750
760
770
780
790
800
810
Consensus
Identity
1. AtSR3
2. AtSR6
3. SlSR3
4. SlSR3L
5. AtSR5
6. SlSR2
7. SlSR2L
8. SlSR4
9. AtSR1
10. SlSR1
11. SlSR1L
12. AtSR2
13. AtSR4
820
830
840
850
860
870
880
890
900
Consensus
Identity
1. AtSR3
2. AtSR6
3. SlSR3
4. SlSR3L
5. AtSR5
6. SlSR2
7. SlSR2L
8. SlSR4
9. AtSR1
10. SlSR1
11. SlSR1L
12. AtSR2
13. AtSR4
Ankyrin Repeats

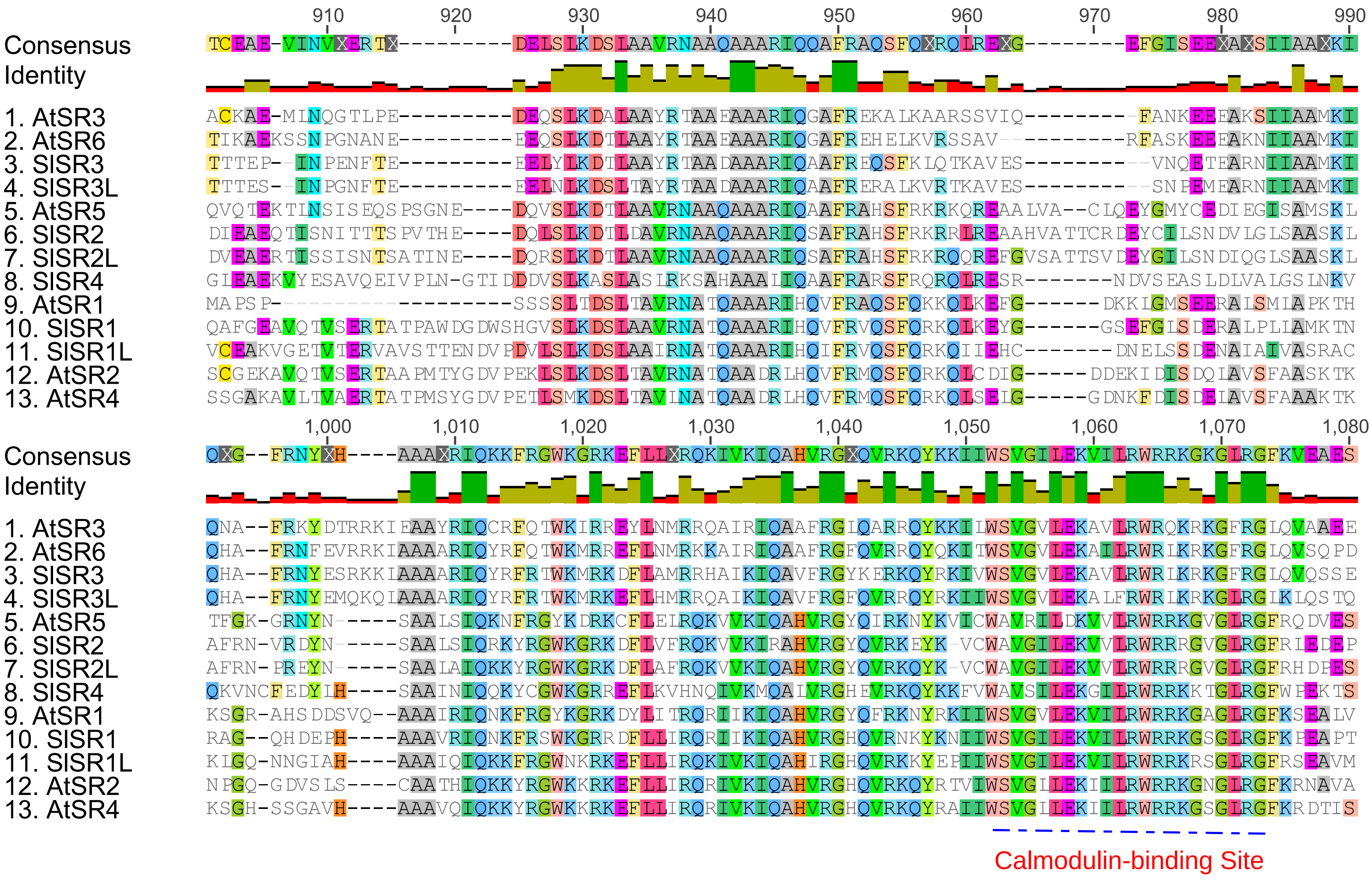

Consensus
Identity
1. AtSR3
2. AtSR6
3. SlSR3
4. SlSR3L
5. AtSR5
6. SlSR2
7. SlSR2L
8. SlSR4
9. AtSR1
10. SlSR1
11. SlSR1L
12. AtSR2
13. AtSR4
910
920
930
940
950
960
970
980
990
1,000
1,010
1,020
1,030
1,040
1,050
1,060
1,070
1,080
Calmodulin-binding Site

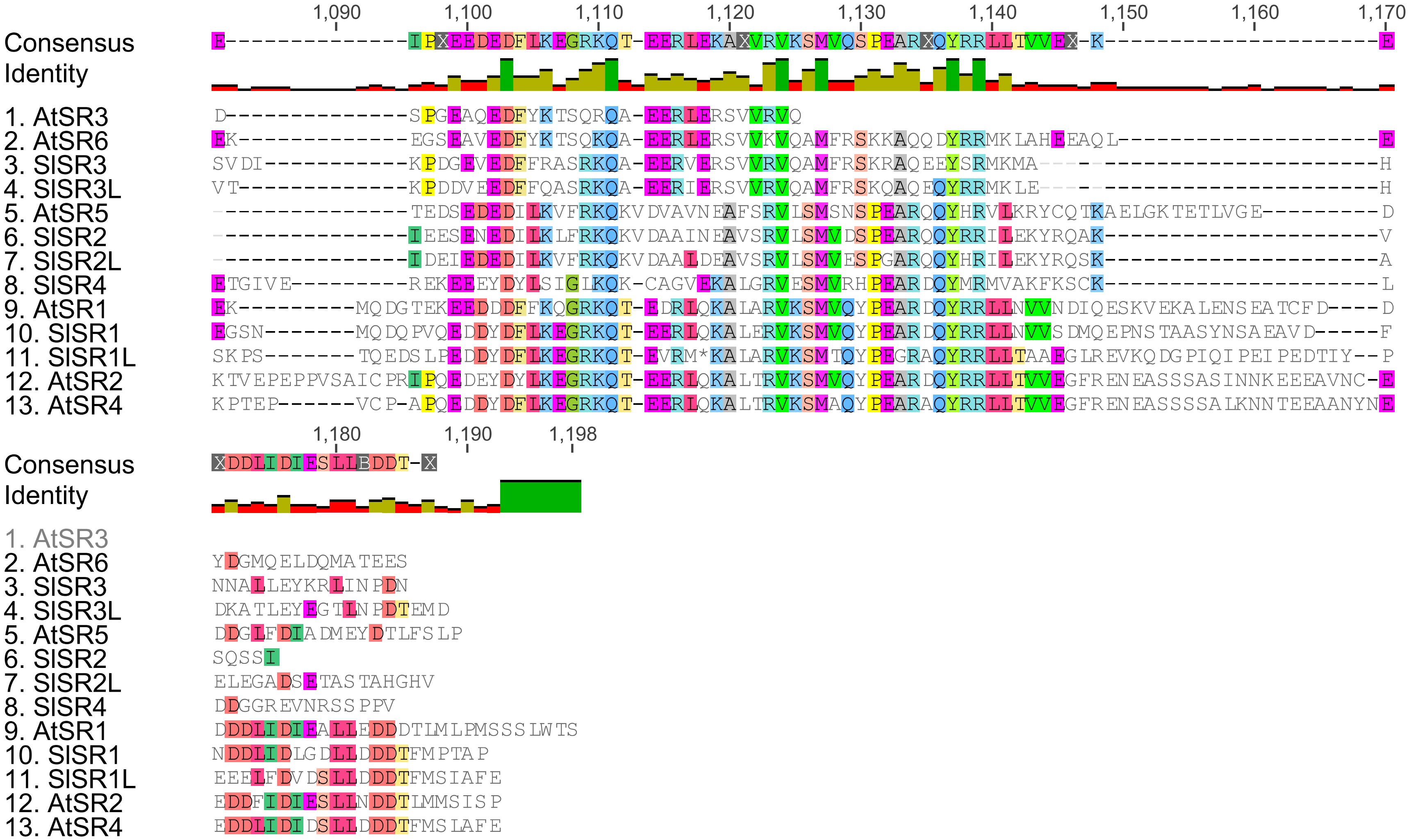
1,090
1,100
1,110
1,120
1,130
1,140
1,150
1,160
1,170
Consensus
Identity
1. AtSR3
2. AtSR6
3. SlSR3
4. SlSR3L
5. AtSR5
6. SlSR2
7. SlSR2L
8. SlSR4
9. AtSR1
10. SlSR1
11. SlSR1L
12. AtSR2
13. AtSR4
1,180
1,190
1,198
Consensus
Identity
1. AtSR3
2. AtSR6
3. SlSR3
4. SlSR3L
5. AtSR5
6. SlSR2
7. SlSR2L
8. SlSR4
9. AtSR1
10. SlSR1
11. SlSR1L
12. AtSR2
13. AtSR4
