# Supplementary figures and images for: Characterization of a calcium/calmodulin-regulated SR/CAMTA gene family during tomato fruit development and ripening
Source: BMC Plant Biol. 2012 Feb 13;12:19. doi: 10.1186/1471-2229-12-19 (PMC3292969; doi:10.1186/1471-2229-12-19)

**S1SR1      WSVGILEKVILRWRRKGSGLRG**

**S1SR2      WAVGILEKVVLRWRRRGVGLRG**

**S1SR4      WAVSILEKGILRWRRKKTGLRG**

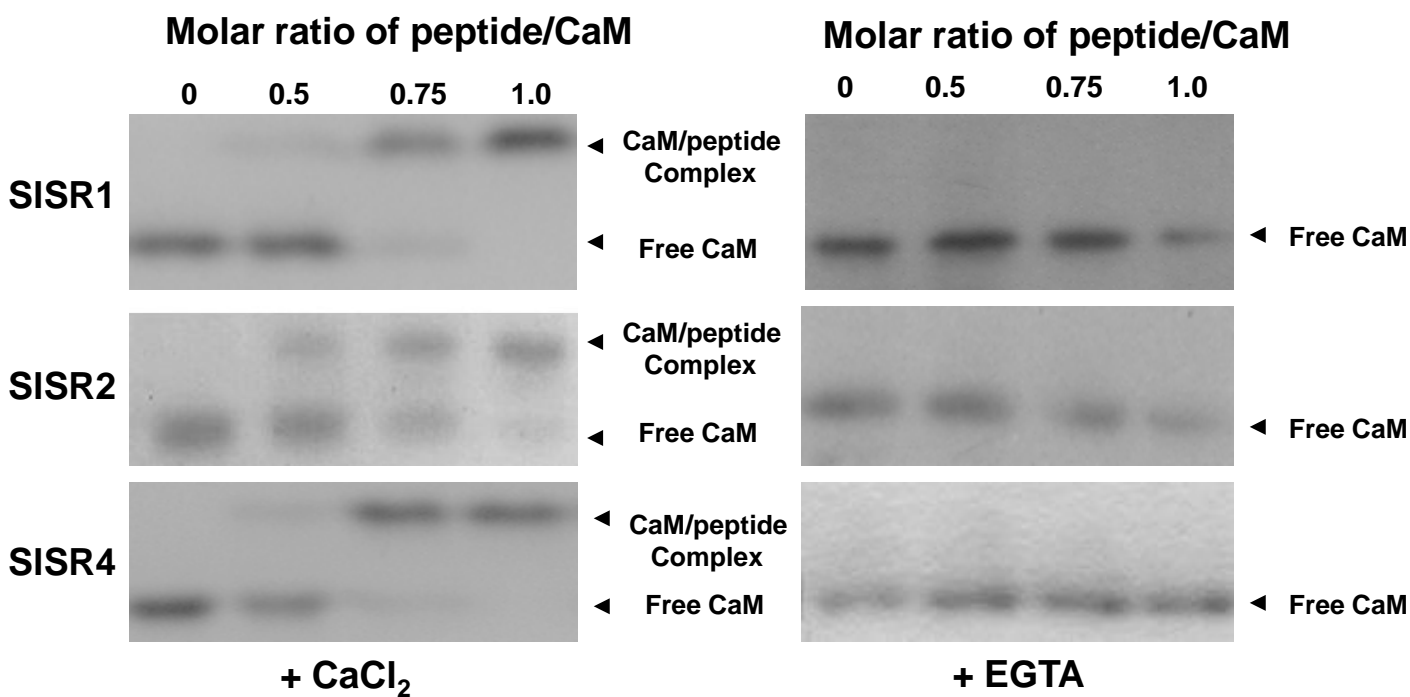

Supplement: Additional file 2 — Calcium/calmodulin binding to peptides from SlSR1, SlSR2/SlSR2L, and SlSR4. Three peptides corresponding to the putative calmodulin-binding domains of SlSR1, SlSR2/SlSR2L, and SlSR4 were synthesized. Gel mobility shift assay showing that calcium-loaded calmodulin binds to all three peptides in the presence of calcium. Arrows indicate the positions of free calmodulin (CaM) and the peptide-calmodulin complexes. [file 1471-2229-12-19-S2.PDF]
